# DNA metabarcoding and microscopic analyses of sea turtles biofilms: Complementary to understand turtle behavior

**DOI:** 10.1371/journal.pone.0195770

**Published:** 2018-04-16

**Authors:** Sinziana F. Rivera, Valentin Vasselon, Katia Ballorain, Alice Carpentier, Carlos E. Wetzel, Luc Ector, Agnès Bouchez, Frédéric Rimet

**Affiliations:** 1 UMR CARRTEL, INRA, Université Savoie Mont-Blanc, Thonon, France; 2 Agence Française pour la Biodiversité, Parc naturel marin de Mayotte, 14 lot. Darine Montjoly, Iloni, Mayotte, France; 3 Association Escale, 13 bis rue Foundi Madi Attoumani, Mronabeja, Mayotte, France; 4 LIST, 41 rue du Brill, Belvaux, Grand-duchy of Luxembourg; University of Hyogo, JAPAN

## Abstract

Sea turtles are distributed in tropical and subtropical seas worldwide. They play several ecological roles and are considered important indicators of the health of marine ecosystems. Studying epibiotic diatoms living on turtle shells suggestively has great potential in the study of turtle behavior because diatoms are always there. However, diatom identification at the species level is time consuming, requires well-trained specialists, and there is a high probability of finding new taxa growing on turtle shells, which makes identification tricky. An alternative approach based on DNA barcoding and high throughput sequencing (HTS), metabarcoding, has been developed in recent years to identify species at the community level by using a DNA reference library. The suitabilities of morphological and molecular approaches were compared. Diatom assemblages were sampled from seven juvenile green turtles (*Chelonia mydas*) from Mayotte Island, France. The structures of the epibiotic diatom assemblages differed between both approaches. This resulted in different clustering of the turtles based on their diatom communities. Metabarcoding allowed better discrimination between turtles based on their epibiotic diatom assemblages and put into evidence the presence of a cryptic diatom diversity. Microscopy, for its part, provided more ecological information of sea turtles based on historical bibliographical data and the abundances of ecological guilds of the diatom species present in the samples. This study shows the complementary nature of these two methods for studying turtle behavior.

## Introduction

Sea turtles play several ecological roles such as maintaining the health of seagrass beds and coral reefs; they also provide habitats for marine organisms, aid in maintaining balanced marine food webs, and promote nutrient cycling from marine to terrestrial ecosystems [1–4]. They are considered important indicators of the health of marine ecosystems [2, 5, 6]. In spite of this, turtle populations have declined significantly as consequence of human activities (e.g., habitat destruction, ocean pollution, poaching, and fishing) [7] and climate change [8]. Nowadays, six of the seven existing species of sea turtles are classified as threatened or endangered by the International Union for Conservation of Nature. Understanding sea turtle behavior (e.g., migration, feeding, and reproduction patterns) is important for their conservation and management [9, 10]. Several methods, such as aerial survey [10–14], snorkeling survey [14, 15] and telemetry [16] (e.g., very high frequency telemetry, sonic telemetry and satellite telemetry), have been used to study and monitor the population and distribution of sea turtles. Data acquisition systems such as GPS tracking, geolocating tags, time-temperature-depth recorders, and heart rate counters were also been used to monitor their behavior and physiology [16–20]. Another method suggestively having great potential for studying turtle behavior is to study the epibiota on shell turtles. Most of these studies have focused on macro-epibiota but, since the 70s (e.g., [21–25]), micro-epibiota has also received attention and is now the subject of growing interest given the diversity of organisms that live on(e.g., [26–28]). Among the micro-epibiotic organisms, diatoms (Bacillariophyta) receive the most attention because of their diversity and density on turtle shells [29–31]. Diatoms are a clade of microalgae ranging in size from a few to several hundreds of micrometers. Their most remarkable feature is their intricately ornamented siliceous exoskeleton, called frustule, which is used to characterize the various species, of which there is an estimated 100,000 species [32]. Recently, several new species have been described from marine [33–38] and freshwater [39–41] turtle shells. The capacity for diatom assemblages to change their species composition with changes in environmental conditions such as water turbulence [42], light intensity [43], and nutrient levels [44] is one of their characteristics.

These properties and their ubiquity make diatoms excellent ecological indicators. They are used worldwide to evaluate the ecological quality of rivers and lakes [44] and, more recently, of costal marine environments (e.g., [45]). They are ever-present on turtle shells [28]. Moreover, the species composition of epibiotic diatoms from freshwater turtles of the same species can differ based on turtle activities and their region of origin [46]. Similar differences have been observed with marine turtles [27]. Nevertheless, such differences were not observed between freshwater turtles (*Emys orbicularis*) coming from various ponds in South France (e.g., [47]) nor for certain marine turtles (*Lepidochelys olivacea*, [28]). Nevertheless, some authors acknowledged that their results should be confirmed [28]. For all these reasons, several researches have increasing diatom potential for studying turtle behavior [28].

Even if diatoms are excellent ecological indicators, the identification of individuals at the species level relies on the morphological criteria of their siliceous exoskeletons. This is time consuming, requires well-trained specialists spending hours under the microscope to establish floristic lists (usually a floristic list is based on 400 identified exoskeletons), and difficulties often appear when differentiating between morphologically near species (e.g., [48, 49]). Moreover, when working on diatom samples such as those of turtles shells, it is highly probable that new taxa to science will be observed, increasing the difficulty in identifying diatoms. In recent years, an alternative approach for identifying diatom species in environmental samples based on DNA has been developed. Metabarcoding [50] uses molecular techniques at the community level by combining DNA barcoding [51] with high throughput sequencing (HTS). DNA barcoding allows an accurate identification of an organism to species level from a short DNA fragment while HTS allows millions of DNA fragments from many samples to be sequenced simultaneously. Sequencing data is then used to obtain an accurate identification of diatom taxa at the species level by comparing to a DNA reference library. Several studies have already shown that metabarcoding has the potential to identify diatoms in freshwater [52–54]. The advantage of metabarcoding over microscopy in the identification of diatom samples is this high throughput.

The aim of this study was to determine whether metabarcoding carried out on epibiotic diatoms from sea turtle shells provide the same information about turtle behavior as does microscopy. In particular, we formed the following hypothesis:

Based on the composition of epibiotic diatom assemblages, turtles can be regrouped in a similar way using either microscopy or metabarcoding. If this hypothesis is correct, what information about the turtle behavior can epibiotic diatoms bring? If it is not correct, several additional questions are raised. Do both methods taxonomically identify the epibiotic diatoms with the same accuracy? Are diatom assemblages obtained with microscopy the same as those obtained with metabarcoding? Does the two methods discriminate epibiotic diatom assemblages equally well? Is there a cryptic diversity (which is difficult to observe in microscopy) that explains differences between microscopy and metabarcoding analyses?

Such questions were addressed for a population of seven juvenile green sea turtles, *Chelonia mydas*, feeding on a seagrass bed from the third largest coral atoll of the world, which is situated near the French Island Mayotte (Comoros archipelago) in the Northern Mozambique Channel.

## Materials and methods

### Sampling

**Fig 1:**
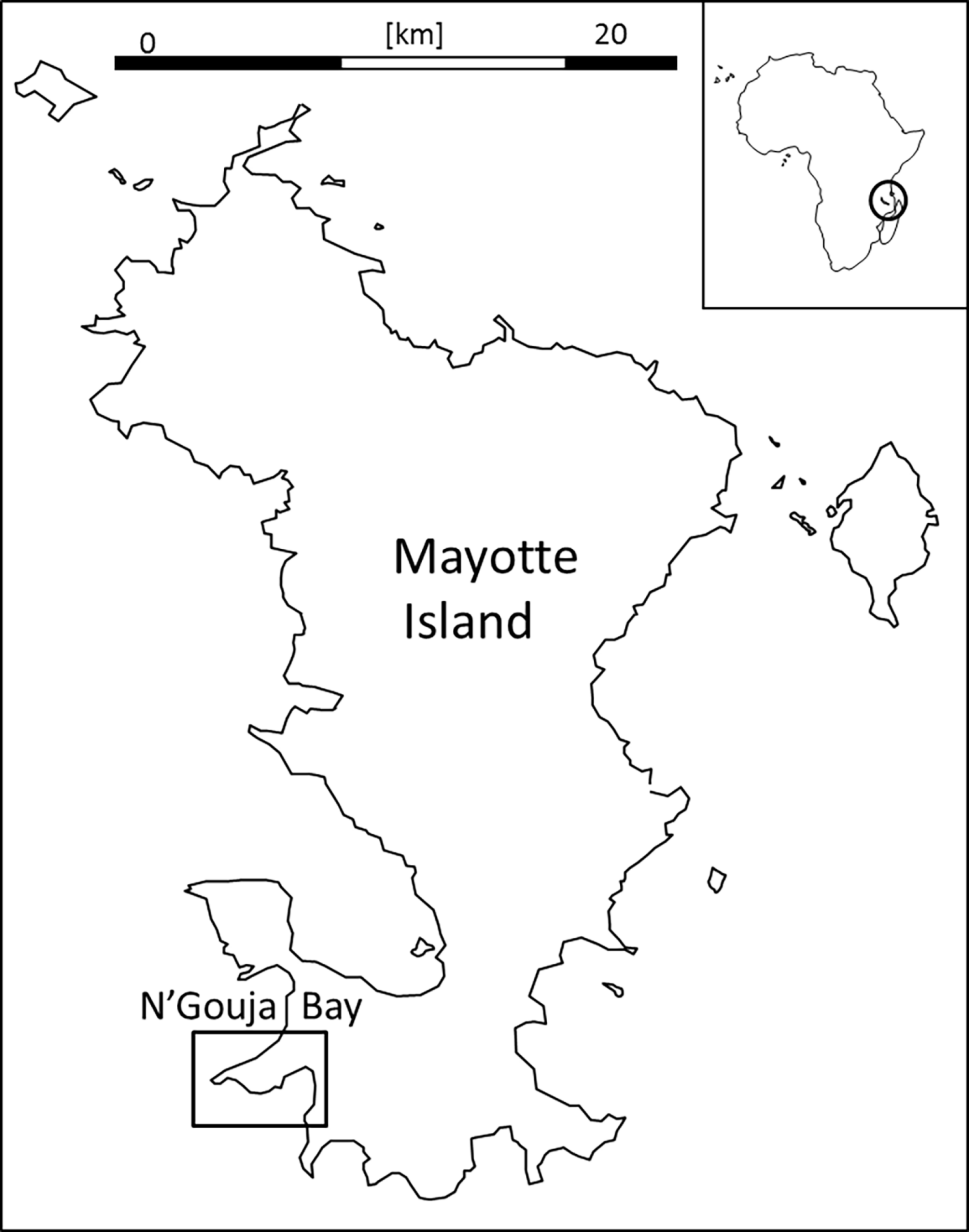
Location of Mayotte Island (France) in the south west Indian Ocean. The black square indicates where the *Chelonia mydas* were sampled.

Epibiotic samples used in this study were collected on 12 October 2015 from seven living green turtles *Chelonia mydas* at N'Gouja Bay in the Marine Nature Park of Mayotte, France (12°57’ 43.05” S, 45°5’ 6.49 E) (Fig 1). The entire shell of the turtle was scraped off using a clean toothbrush. This protocol was non-invasive and was limited to the external part of the turtle shell, and it does not harm or cause the animal suffering. All the procedures involved respect the ethical standards in the Helsinki Declaration of 1975, as revised in 2000 and 2008, as well as the applicable national law. All sampling procedures were carried out by A. Carpentier who was authorized by the Mayotte Prefecture to capture turtles and sample the epibiotic organisms.

Samples were fixed in ethanol (70% final concentration) according to European protocols [55] and kept cold (4–7°C) until molecular and microscopic treatments. Tag and physical details for each turtle are presented in Table 1.

**Table 1:** Details of the turtles sampled for this study.

Turtle	Name	Age class	Tag left fin	Tag right fin	CCL [cm]	SCL [cm]	Weight [kg]
1	Zorro	Juvenile	yt5990A	yt4033B	71.0	66.0	41.40
2	Efe	Juvenile	yt4016B	yt5901A	62.5	57.0	29.18
3	Lost	Juvenile	yt4015B	yt5949A	56.0	52.0	18.91
4	Uhu	Juvenile	yt4019B	yt5943A	58.0	54.0	21.96
5	Mabawa	Juvenile	yt4020B	yt5945A	74.0	68.0	45.40
6	Digueline	Juvenile	yt4032B	yt5950A	74.5	68.5	48.00
7	Bertha	Juvenile	yt4021B	—	73.5	69.0	46.26

CCL, curved carapace length; SCL, straight carapace length

### Light and scanning electron microscopic analyses

Samples were cleaned with 40% H_2_O_2_ and HCl according to European standard EN 13946 [56]. After repeated rinsing and decantation with distilled water, air-dried aliquots were mounted on permanent glass slides using Naphrax®. At least 400 valves were identified and counted under the light microscope at a magnification of 1000X using a Zeiss Axio Imager A1® microscope according to European standard EN 14407 [57]. Taxonomic identifications were performed based on the specific marine diatom floras of [58–60], and also on the flora of [61]. Other papers dealing on marine species were also used (e.g., [62–64]). A list of the taxa and their relative abundances was produced for each of the 7 samples.

Scanning electron microscopic examinations were carried out on an ultrahigh-resolution analytical field emission (FE) scanning electron microscope Hitachi® SU–70 (Hitachi High-Technologies Corporation, Japan) using an accelerated voltage of 5 kV. For these examinations, cleaned oxidized samples were concentrated on a polycarbonate membrane filter with a 3-μm mesh, attached to aluminum stubs, and sputtered with a 30-nm platinum layer.

In order to answer the question about the cryptic diversity of *Nitzschia inconspicua* Grunow, light and scanning electron microscopic photos were taken.

### Molecular analysis

DNA extraction, PCR amplification, sample libraries preparation, and HTS followed the technical specifications given in [65]. Briefly, DNA extraction was based on Sigma-Aldrich GenElute™-LPA DNA precipitation, and PCR amplification was performed on the *rbc*L plastid gene, targeting a 312 bp barcode. To amplify the entire diatom diversity, equimolar mixes of 3 forward and 2 reverse primers were used as described previously [66]. For each DNA sample, PCR amplification was carried out in triplicate. To prepare sample libraries, the PCR products of each triplicate were pooled. After cleaning and checking for DNA purity and quantity, tags were added to each amplicon. Library preparation was performed as described in [65]. Libraries were sequenced on a PGM Ion Torrent machine by the “Plateforme Génome Transcriptome” (PGTB, Bordeaux, France) using the Ion 318™Chip Kit V2 (Life Technologies, Carlsbad, USA) on 7 December 2016.

Bioinformatic processing was performed according to the technical specifications given in [65]. In brief, after several steps of quality filtering, DNA reads were clustered into Operational Taxonomical Units (OTUs) using a distance similarity threshold of 95%. For each sample, a list of OTUs and their numbers of reads were obtained. Taxonomical identifications of the OTUs were obtained using the R-Syst::diatom library [67] (R-Syst::diatom v4, of 16-09-2015, http://www.rsyst.inra.fr/en).

### Statistical analyses

Statistical analyses were carried out using XLSTAT version 2011.4.04 Addinsoft^TM^.

To test our hypothesis that based on the composition of epibiotic diatom assemblages, turtles can be regrouped similarly with microscopy and metabarcoding, a K-means clustering (using Bray-Curtis distances) was applied on the seven species lists (one for each sample), obtained using microscopy. The same was applied on the seven OTU lists, obtained using metabarcoding. For this statistical analysis (K-means), and those that followed, the OTUs and species were expressed in relative abundances (in percentages) for each sample. Given the number of samples, 3 groups were defined. Moreover, to visualize the epibiotic diatom assemblages, NMDS based on Bray-Curtis distances were drawn using XLSTAT software.

To determine whether the two methods discriminate epibiotic diatom assemblages equally well, a Student t test was performed to compare the Bray-Curtis distances calculated for the diatom assemblages obtained using microscopy with those obtained using metabarcoding.

To determine whether structure of diatom assemblages obtained using either method is the same, distance matrices (Bray-Curtis distances) were calculated between the species list (obtained using microscopy) and the OTU list (obtained using metabarcoding) for each sample. A Mantel test was then used to test the correlation between matrices.

If these three tests (K-means clustering, comparisons of Bray-Curtis distances, and the Mantel test) would show that both microscopical and molecular methods gave different results, then tests for cryptic diversity would be necessary. To this end, one of the most abundant species will be selected for carrying out further tests. A phylogeny will be calculated using reference sequences from R-Syst::diatom [66]. Then OTUs sequences corresponding to this species will be constrained by this phylogeny (see next section for detailed explanations), and co-occurrence in the samples of these OTUs will be tested. Correlations (Spearman coefficients) between the abundance of each OTUs in each samples would be calculated. If not significant, morphologic cryptic diversity can be argued, and metabarcoding can be used to detect cryptic diversity, whereas microscopy cannot. This would explain the differences between microscopy and metabarcoding in terms of community structure.

### Phylogenetic analyses

To visualize the phylogenetic position of the 100 most abundant OTUs in the diatom phylogeny, a constraint phylogeny was carried out as explained in Rimet *et al*. [68]. Briefly, turtles OTU sequences were aligned with all the *rbcL* sequences in the R-Syst::diatom library using Muscle [69] in Seaview [70]. The best substitution model was then tested in MEGA7 [71]. A constraint phylogeny was then calculated in raxmlGUI [72] using the fast tree search option. The constraint sequences were the turtle OTUs sequences and the shortest sequences of R-Syst::diatom library. A tree was drawn using the online tool iTOL [73] (https://itol.embl.de).

A second phylogeny was carried out on *Nitzschia inconspicua* sequences from the R-Syst::diatom library and their neighbor OTU in the raxmlGUI software using the maximum likelihood and the thorough bootstrap option (see former section). A phylogeny was drawn using Mega7 [71].

## Results

### Microscopic analyses

**Fig 2:**
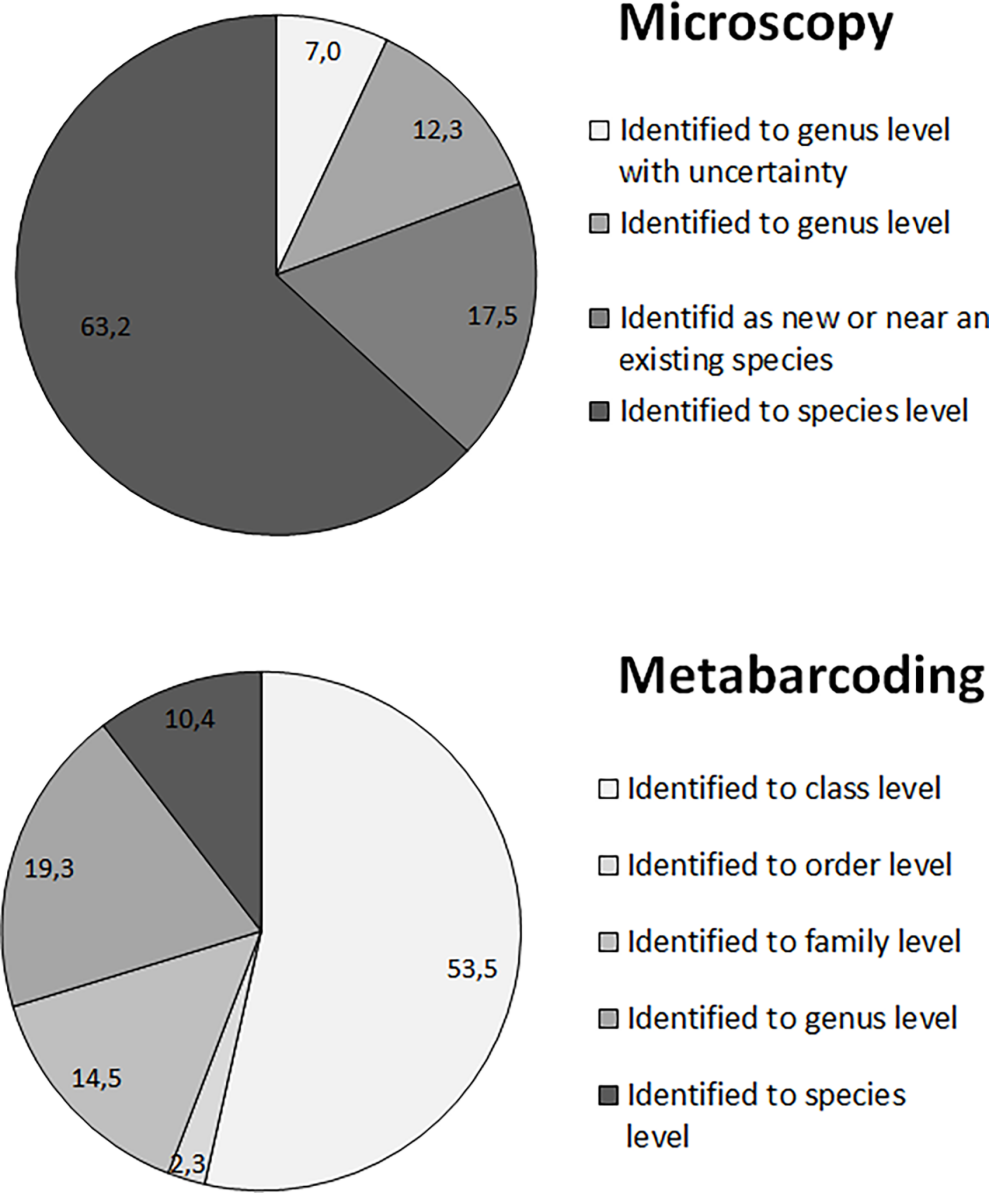
Identification levels reached by microscopy and by metabarcoding. Percentages of the identified taxa through microscopy and of the OTUs identified using R-Syst::diatom v4 (version of 16-09-2015) are given in the pie charts, respectively.

In total, 57 taxa were identified. The number of taxa per sample ranged from 15 to 24 with an average of 20 taxa per turtle. The most abundant taxon (average abundance across all the samples: 51%) was a small *Labellicula* (4 to 6 μm long and 1–1.5 μm wide) corresponding to the recently-described *L*. *lecohuiana* Majewska & Van de Vijver from sea turtles in Costa Rica. For this species, identification was possible only through electron microscopy because almost no visible features were observable using light microscopy. The second-most abundant species (average abundance across all samples: 14%) was *Nitzschia inconspicua* Grunow, a worldewide spread benthic euryhaline species. The third was *Halamphora tenerrima* (Aleem & Hustedt) Levkov (6%), also a widely distributed benthic marine species. For most (63%), species-level identification was reached (Fig 2), but an important part (37%) could not be identified precisely. Many of these taxa might be new to science.

**Fig 3:**
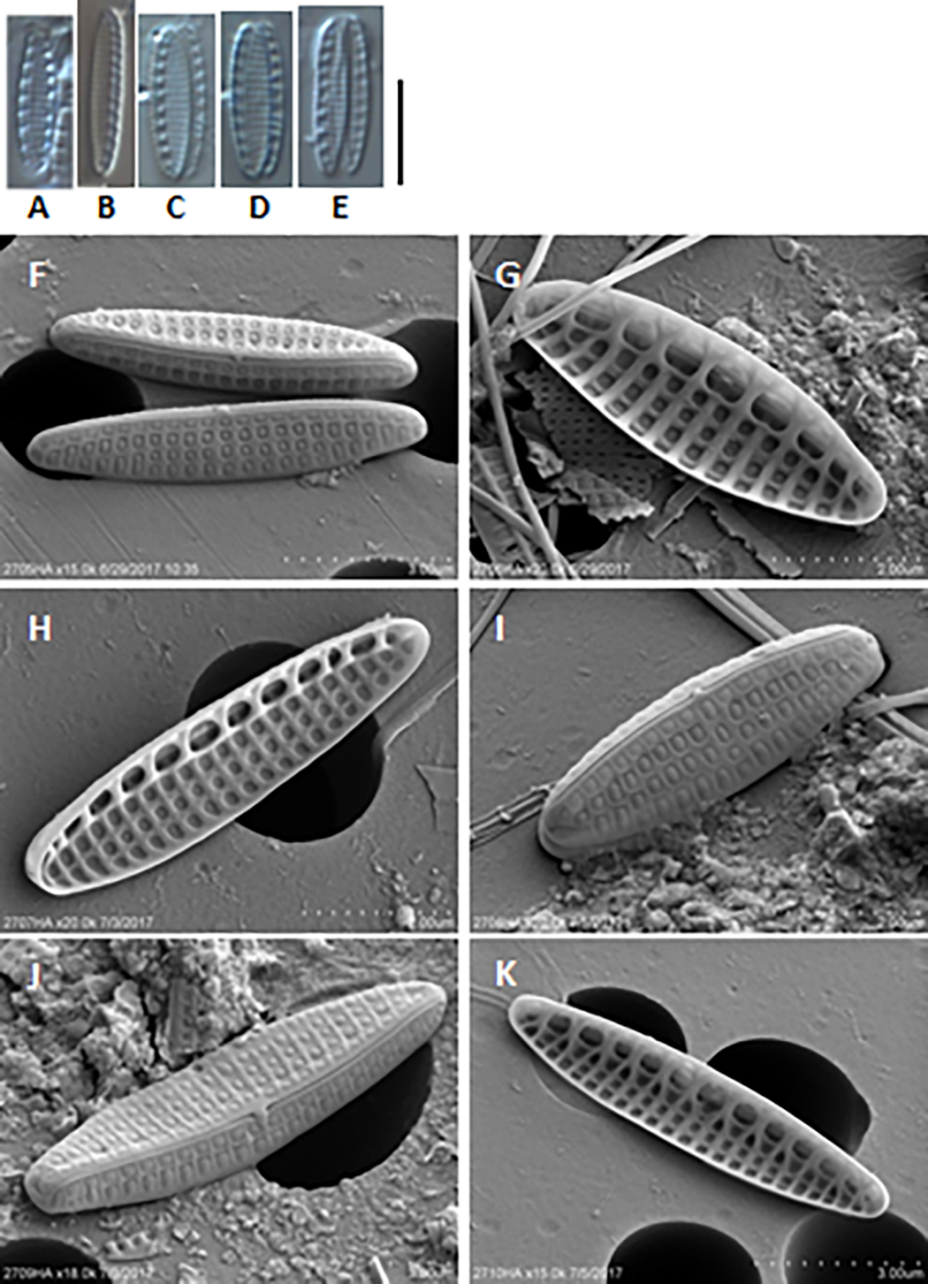
Light and scanning electron microscopy images of *Nitzschia inconspicua* from each turtle. **A―E**. Light microscopy. **A.** Turtle 1. **B**. Turtle 2. **C―D**. Turtle 5. **E.** Turtle 6.**F―K**. Scanning electron microscopy. **F**. Turtle 1. **G.** Turtle 2. **H.** Turtle 3. **I**. Turtle 4. **J**. Turtle 5. **K.** Turtle 6. Scale bar: 10 μm.

Fig 3 presents light and scanning electron microscopy photos of *Nitzschia inconspicua* from several turtles. The morphology of this species is homogeneous across all seven turtles.

### Metabarcoding analyses

In total, 510,922 reads were obtained, averaging 72,989 reads per sample. After the various curation steps (i.e., length, quality, chimera, and alignment), 209,095 reads were conserved, averaging 29,871 reads per sample. Clustering reads at the 95% level resulted in 634 OTUs (range, 231–360 OTUs per sample; mean OTU in a sample, 280).

Taxonomic assignment of the OTUs using the R-Syst::diatom database v4 (16-09-2015) resulted in the identification of 19 species and 26 genera. Only 10% of the 634 OTUs could be assigned at the species level, 19% at the genus level, and 14,5% at the family level (Fig 2). More than 50% were identified at the class level only (diatoms are composed by 4 classes: Bacillariophyceae, Coscinodiscophyceae, Fragilariophyceae, and Mediophyceae).

### Statistical analyses

**Fig 4:**
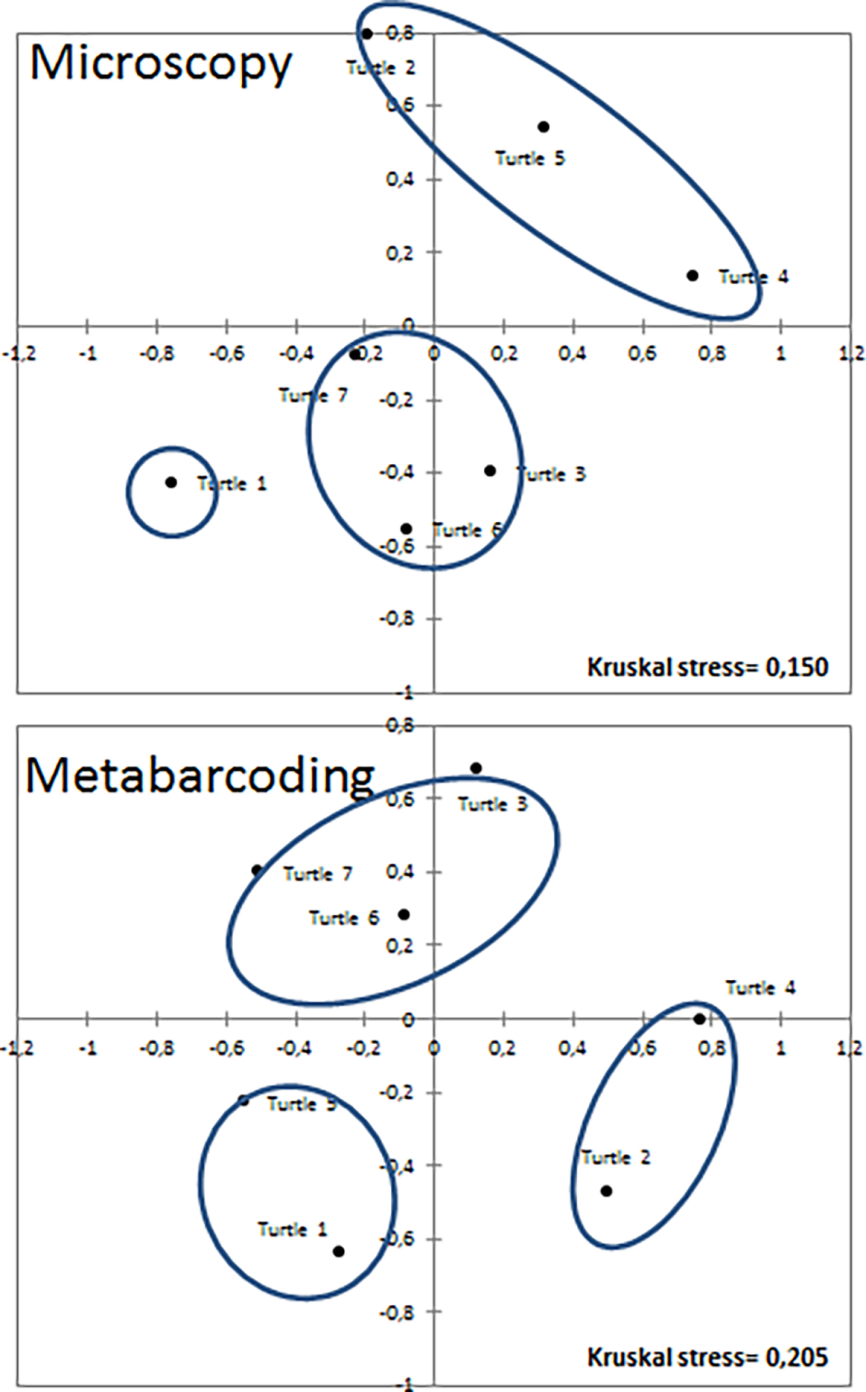
NMDS ordination plots and cluster representations. K-means and NMDSs were calculated based on the Bray-Curtis distances of the species lists and the OTU lists (each expressed in percentages) obtained using microscopy and metabarcoding, respectively.

K-means clustering and NMDS were carried out on the species lists established using microscopy and on the OTU lists established using metabarcoding. Cluster results (Fig 4) differed between both methods; therefore, we must reject our initial hypothesis and concluded that, based on the composition of epibiotic diatom assemblages, turtles cannot be regrouped similarly using microscopy versus metabarcoding.

The Mantel test carried out between the Bray-Curtis distance matrix of the microscopic species lists and the Bray-Curtis distance matrix of the OTUs lists was non-significant (p-value: 0.08), even though the distances matrices tented to be correlated (r^2^ = 15%). Based on these results, the structure of epibiotic diatom is not revealed in the same way by microscopy and by metabarcoding. Moreover, the Bray-Curtis distances revealed that we metabarcoding is significantly better than microscopy for discriminating diatom communities (0.55 vs. 0.27, p-value < 0.001, Student t test).

### Phylogenetic analyses

**Fig 5:**
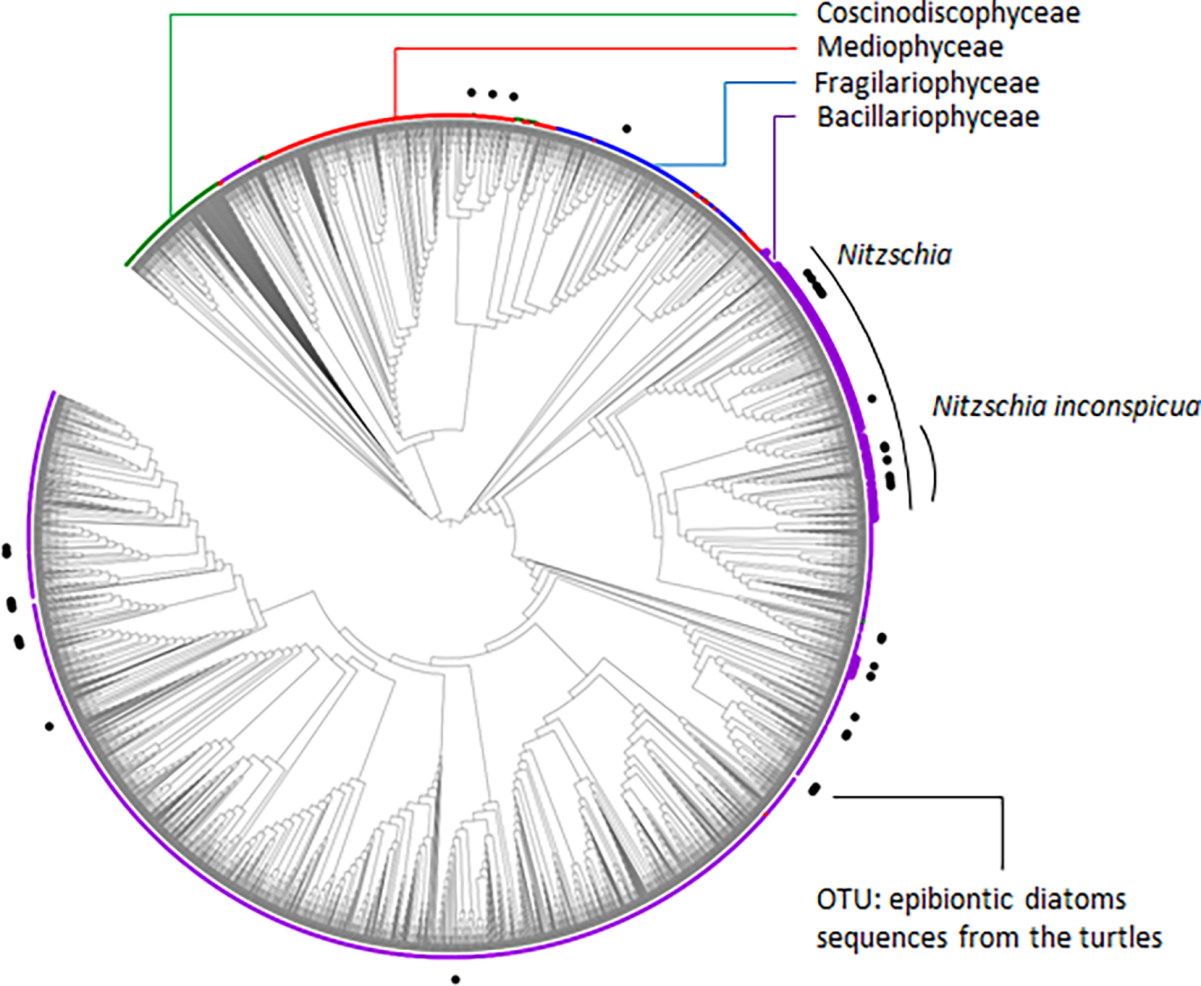
Constraint phylogeny based on R-Syst::diatom sequences and on the OTU sequences of samples taken from the shells of sea turtles. This tree was obtained using the fast tree search option in RaxML and was drawn using iTOL [73] http://itol.embl.de.

A constraint phylogeny was calculated using the fast tree search option in RaxML (Fig 5). In total, 2859 *rbc*L sequences were used in the alignment, and 813 were constrained among which the 100 most abundant OTU of the turtles samples. Phylogeny was carried out on 1532 nucleotides, using a GammaGTR substitution matrix (GAMMA rate heterogeneity model, ML estimate of the alpha-parameter). Most identified OTU were from the Bacillariophyceae family or of the *Nitzschia* genus. The species encompassing the highest number of OTUs was *Nitzschia inconspicua* (sensu lato).

**Fig 6:**
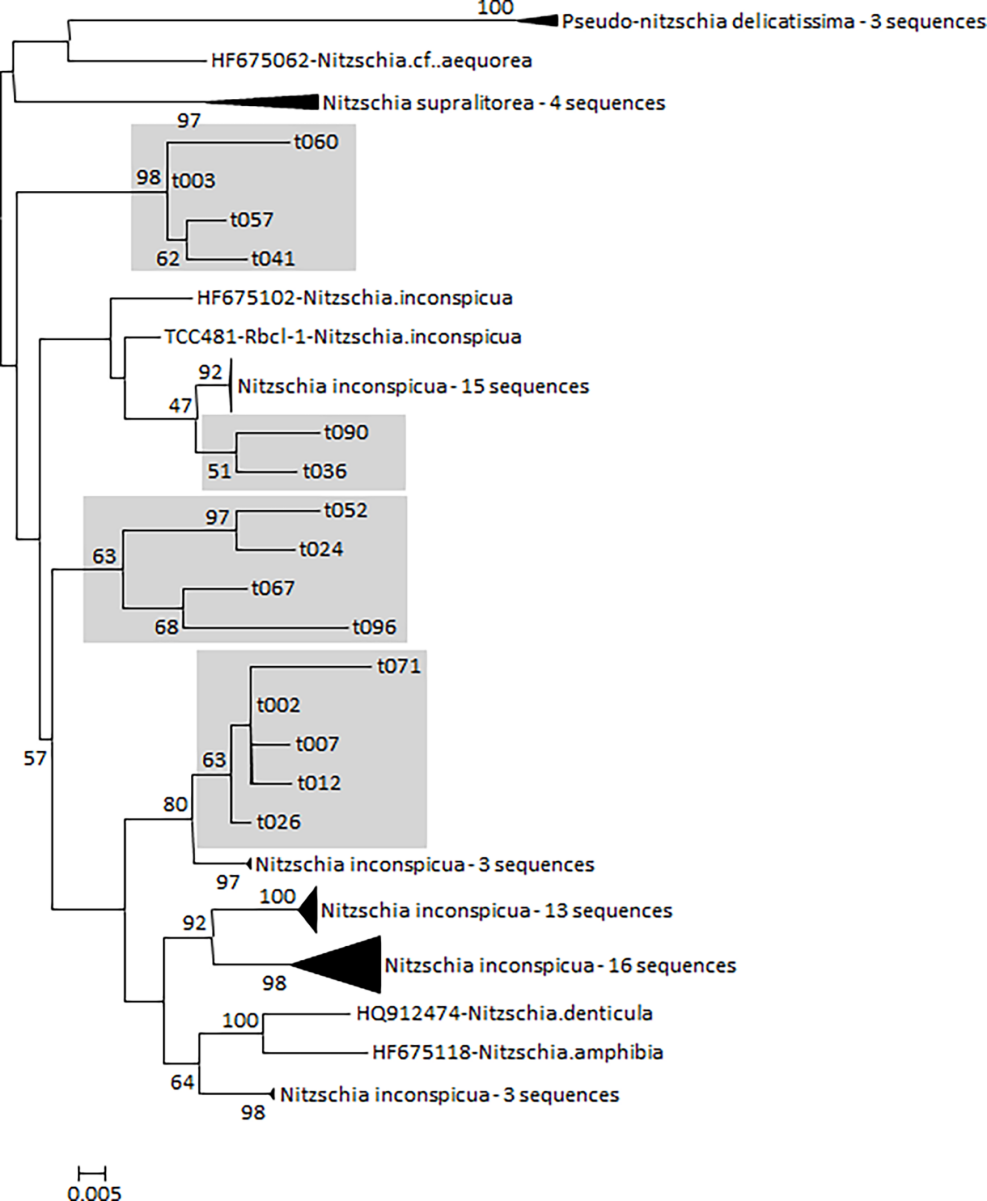
Constraint phylogeny based on *Nitzschia inconspicua* sensu lato and its neighboring species from R-Syst::diatom sequences and on the neighboring OTU sequences of samples taken from the shells of sea turtles. This tree was obtained using the rapid bootstrapping option in RaxML and was drawn using MEGA7. Only the OTUs were constrained. Grey squares delimit four groups of OTUs that co-occurred on the same turtles.

*N*. *inconspicua* was the species chosen for testing cryptic diversity because it is the second most abundant species observed in microscopy and because it encompasses the greatest number of OTUs. Even though *Labellicula lecohuiana* was the most abundant species observed in microscopy, it was not selected because its phylogenetic position is unknown. A detailed constraint phylogeny of *Nitzschia inconspicua* and its neighbor species (*N*. *supralitorea* Lange-Bertalot, *N*. *amphibia* Grunow, *N*. *denticula* Grunow) as well as the neighboring OTU was calculated using RAxML and the rapid bootstrapping and subsequent ML search option (Fig 6). *Pseudo-nitzschia delicatissima* (Cleve) Heiden was used to root the tree. The tree was drawn using MEGA7 [71]. The alignment used for this phylogeny is given in S1 Table. Four well-supported groups of OTUs are revealed by this phylogeny; three are embedded in *Nitzschia inconspicua* sensu stricto, and on its periphery.

**Fig 7:**
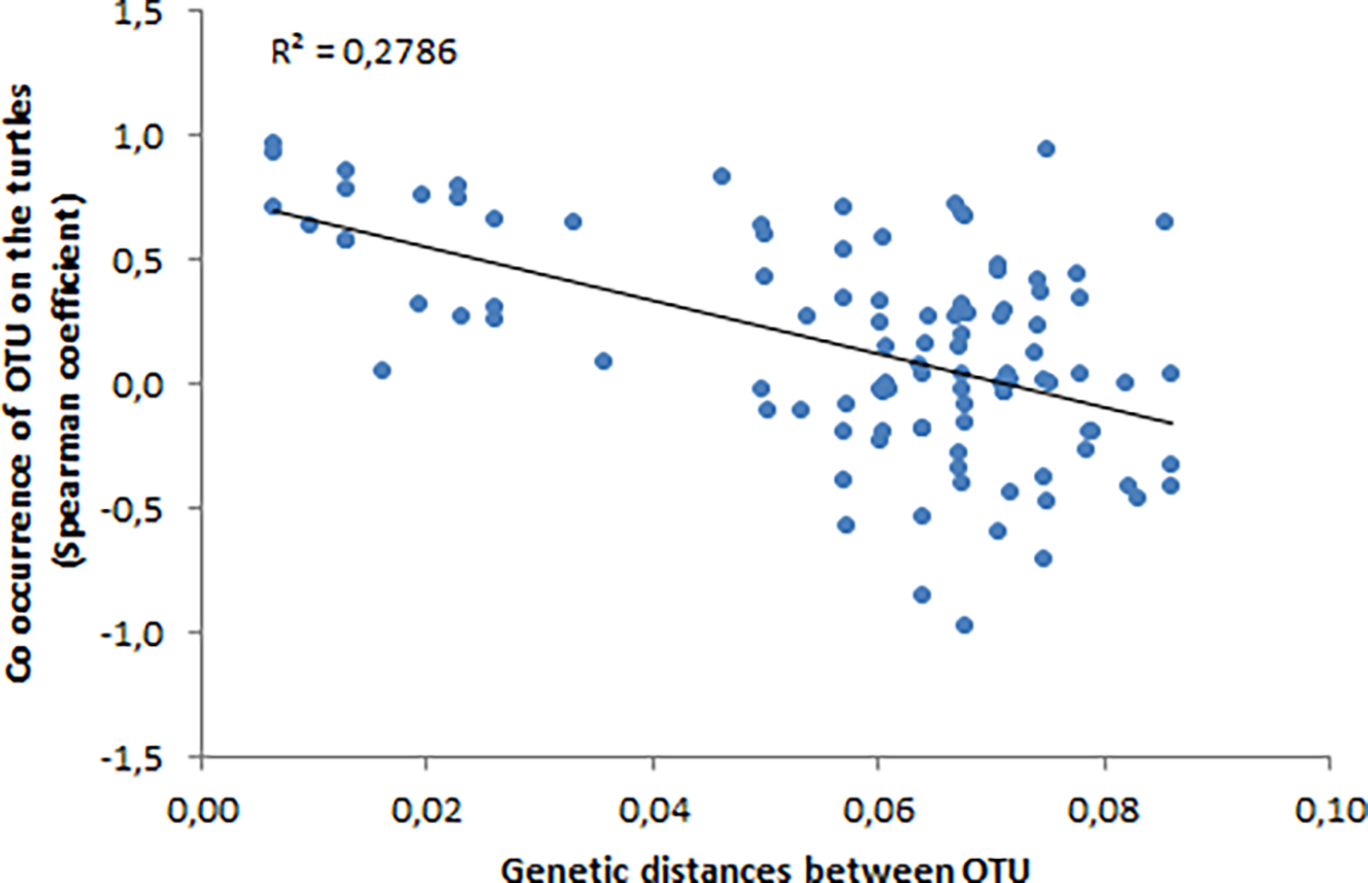
Relationship between the genetic distances of OTUs and their occurrences on sea turtles. **Genetic distance is based on the number of base substitutions per site between sequences**. Occurrence is based on the Spearman correlation coefficient calculated for the abundance of OTUs in the samples. The relationship is linear and significant (p-value < 0.001).

To determine whether these OTUs occurred on the same turtles, correlation coefficients (Spearman) between OTUs and their abundances in the samples were calculated. Results show that some OTUs always occur on the same turtles, but others do not (see S2 Table). We hypothesized that genetically similar OTUs occurred on the same turtles, signaling a relationship between genetic distance and the occurrence of these OTUs in the samples. To test this hypothesis, genetic distances were calculated using MEGA7, and the number of base substitutions per site between sequences, applying the maximum composite likelihood model. A correlation between the Spearman correlation coefficients (based on OTU abundances) and the genetic distance was tested and is represented in Fig 7. The tendency for OTUs to be genetically similar on the same turtle is significant. On the other hand, when OTUs were genetically different, they were not present on the same turtle. When genetic distances were less than 0.04 substitutions/site, OTUs co-occurred on the same turtle. At greater distances, they did not occur on the same turtle (Fig 7). When OTUs with distances less than 0.04 substitutions/site are clustered, they form four well-supported groups, as shown in Fig 6. This implies cryptic diversity inside *Nitzschia inconspicua*, comprising four groups that do not have similar ecologies.

## Discussion

### Why the epibiotic diatom assemblages on sea turtles differ between metabarcoding and microscopy?

Results of metabarcoding and microscopic analyses are not comparable in several aspects: turtles are clustered differently based on their diatom assemblages, diatom assemblage structures differ, and metabarcoding yields better discrimination between turtles based on their diatom assemblages. This is unusual, because other studies [66, 74] comparing metabarcoding assemblages (expressed in OTUs abundances) with microscopic assemblages (expressed in species abundances) of benthic diatoms showed the methodologies to be similar in terms of diatom assemblages. As these authors, we chose to compare the microscopic assemblages expressed in species abundances to metabarcoding assemblages expressed in OTUs abundances. Metabarcoding is expressed in OTU abundances because expressing assemblages in species abundances, when an important part of the OTUs were not identified; would result in a comparison based on data truncated at least 64% for metabarcoding data (see Fig 2). Using OTU abundances without taxonomic assignation allows all data to be kept [75]. Several reasons can explain the differences between the methodologies.

1. First, a classical statistical problem exists. The number of samples is quite low (only seven turtles were sampled), and this can explain why the structure of epibiotic diatom assemblages obtained with both methods were not the same. The former studies of [74] and [66], obtained correlation factors between both methods of 18% and 20% across 90 samples and 66 samples, respectively, and the methods were found to be significantly correlated. Our results (15% correlation across 7 samples) are in the same range, but are slightly weaker.
2. Second, each methodology measures something different. In microscopy, diatom extracellular skeletons (siliceous frustules) are counted even if they come from dead cells and stay glued to the biofilm without DNA in the sample. Metabarcoding won't detect these dead cells. This phenomenon has already been mentioned by others [52, 53, 66, 74]. Also, in microscopy, a single cell of a small species such as *Labellicula lecohuiana* can have a biovolume of 15 to 20 μm^3^, but has the same importance as a larger species such as *Entomoneis punctulata* (Grunow) Osada and Kobayasi, which has a biovolume of 1500–2000 μm^3^. In microscopy, skeletons are counted in the same way regardless of biovolume. On the other hand, the number of copies of *rbc*L genes in a cell depends on its biovolume [66, 76]. Therefore, for a similar number of large cells in a sample, a greater abundance of OTUs will be found, compared to small species. This has an obvious impact when comparing metabarcoding with microscopic diatom assemblage structures.
3. Third, samples were dominated by many very small species such as *Labellicula* spp. (average length, 5 μm), *Halamphora* spp. (length, 5–10 μm), and unidentified *Navicula* (length, 5–8 μm), all with cell widths around 1–2 μm. Such frustules were very difficult to identify and count precisely under light microscopy, and under scanning electron microscopy, a number of identification uncertainties could not be confirmed. Freshwater samples from Mayotte Island sampled and analyzed in microscopy for routine monitoring [77] and in metabarcoding (e.g., [66]) do not typically have so many small species. This is also the case for freshwater samples on mainland France (e.g., [78]). Identification using light microscopy reaches its limits when small species are too abundant; therefore, microscopic counts introduce significant uncertainty. A large set of epibiotic diatoms samples from turtles were analyzed using scanning electron microscopy, thus bypassing this problem and enabling robust identification [27]. Nevertheless, scanning electron microscopy is expensive and cannot be undertaken routinely. Use of the methodology is exceptional.
4. Fourth, many taxa examinated microscopically were probably new to science and this is a source of difficulty when establishing robust taxonomic lists. It reduces the ability of microscopy to differentiate turtles on the basis of their epibiotic diatom assemblages. More than 36% of the taxa from our samples were not identified with certainty at the species level. It is not unlikely that these unidentified diatoms represent probable new taxa; as many as 10 new taxa found on sea turtles have been described since 2015 [33–38]. In their paper, Majewska *et al*. [79], already observed that many new taxa remain undescribed in epibiotic samples and this is frequent in littoral marine habitats [32]. With metabarcoding, using diatom assemblages based on their OTUs abundances (with no taxonomic assignment) to compare sea turtles enables a much more objective and precise comparison than microscopy, even though metabarcoding includes bias (e.g., choice of the extraction method, and sequencing errors; see [74]). This bias should be the same among all samples.
5. Fifth, cryptic diversity in diatoms has been extensively studied, focusing on a few diatom species, and morphology has been shown to explain only part of the species diversity. Inside an apparently single morphological species, certain reproductive barriers exist, explaining why certain genetic groups exist while sometimes displaying slight morphological differences observables only after careful examination (e.g., *Sellaphora pupula* [80, 81]). This cryptic diversity explains differences between individuals in terms of ecological requirements for apparently cosmopolitan species (e.g., *Pinnularia borealis* and *Hantzschia amphioxys*, [82]). Moreover, these cryptic species can have different ecologies and can live in sympatry. This is the case for *Navicula phyllepta*, a marine species consisting of several cryptic taxa which have different tolerances in terms of salinity but which live in the same tidal flat in the Netherlands [83].

*Nitzschia inconspicua* was one the most abundant taxa observed in the turtles samples from Mayotte. The morphology of this species was homogeneous across the seven turtles. However it was made up of tens of OTUs which could be regrouped into four groups. Inside these four groups, OTUs occurred on a given turtle; conversely, these four groups did not occur together on a given turtle. This implies a cryptic diversity for *N*. *inconspicua*, which is usually described as cosmopolitan and euryhaline [84, 85]. We hypothesize that *N*. *inconspicua* comprises several cryptic taxa whose ecological requirements differ between each other. This could explain the apparent cosmopolitanism of this species as well as others (*Pinnularia borealis*, *Hantzschia amphioxys*, *Sellaphora pupula*, and *Navicula phyllepta*). Likewise, a study on diatom diversity in high altitude lakes [86] raised the hypothesis that HTS could detect cryptic diversity. Our study validate this hypothesis: cryptic diatom diversity is detectable in metabarcoding and surely makes a valuable contribution for the fine scale understanding of turtle behavior in Mayotte. This cryptic diversity also explains why metabarcoding enables better discrimination between sea turtles than microscopy.

### The ecology and architecture of turtle biofilms can be characterized by microscopy, not by metabarcoding

More than 93% of the taxa could be identified at the species or genus level under microscopy. Even if species identifications based on microscopy were doubtful and yielded poor discrimination of turtles based on their epibiotic diatom assemblages, this was sufficient to give a rough but robust idea of the ecology of the diatoms living on these turtles. Sister species of diatoms were shown to typically have the same ecology [87], and that identification at the genus level enables a quick and robust ecological assessment [46, 88, 89]. Moreover, the ecological guilds used can be assigned to most of the taxa if their genera and sizes are known [77]. We could gain this information via microscopy.

From the abundance of ecological guilds, epibiotic diatoms are clearly from a benthic origin and are mostly loosely attached (see Table 2). In short, the turtles could be assumed to be slow mover. Nevertheless, some differences between turtles can be observed. For instance, Turtle 2 presented the lowest abundance of low-profile diatoms (adapted to resist water turbulences); therefore, it was likely a slow swimmer. On the other hand, Turtle 4 presented three times more low-profile diatoms and therefore probably swam faster.

**Table 2:** Abundance of diatom ecological guilds on turtle shells.

Turtle	High profile [%]	Low profile [%]	Motile [%]	Planktonic [%]
1	5.9	12.9	80.7	0.5
2	12.5	5.2	81.2	1.2
3	4.0	13.2	82.8	0.0
4	11.5	17.6	70.0	0.9
5	6.5	9.2	84.3	0.0
6	3.9	14.2	80.0	1.9
7	3.9	12.7	83.5	0.0

High-profile encompasses cells attached to substrate but enable to resist turbulences. Low profile are cells firmly attached to substrate and resisting to turbulences. Motile are loosely attached cells moving in biofilms. Planktonic are free-floating cells.

All the taxa observed on these sea turtle shells were strictly marine. Nevertheless, one dominant species, *Nitzschia inconspicua*, is known to be a marine-brackish euryhaline taxon [90]. This probably reflects the travels of the turtles from water bodies of varying salinities or from various depths, showing salinities gradients in Mayotte [91].

The totality of the diatom taxa observed on these turtle shells are known to be from habitats other than turtles shells [40], except *Tursicola* sp. and *Labellicula lecohuiana*. *Tursicola* sp. is a genus known only from epibiotic habitats [40], but it was rare on Mayotte turtles (observed only on Turtle 1 at an abundance below 1%). *Labellicula lecohuiana* was dominant on all the turtle shells of Mayotte. This is a recently-described species also coming from shells of the same sea turtle species, *Chelonia mydas*, but from Costa-Rica [35]. Therefore, it is known to come only from epibiotic habitats. Nevertheless, the *Labellicula* genus is very closely related to *Olifantiella* (according to Majewska *et al*. [35]), which encompasses several species from the coral sands of various tropical islands [62]. Therefore, it would be necessary to sample the coral sands of Mayotte Island to be assure that *Labellicula lecohuiana* is not present on that substrate, making this species strictly epibiotic. We believe that the epibiotic diatoms living on turtle shells in Mayotte originate from benthic habitats: when turtles graze seagrass, they resuspend sand, which falls on their shells. Therefore, the shells are constantly seeded with benthic diatoms from their surrounding habitats. We believe that the hypothesis that these diatoms are strictly epizoic is weakly probable for the turtles we studied. We believe that diatom composition of the sea turtle shells is related to the diatom composition of its surrounding environment as it has been stated by Majewska *et al*. [27].

All this ecological information could not be gained only from metabarcoding, simply because some references in the R-Syst::diatom barcoding library are lacking. Only 29% of the OTUs could be identified at the species or genus level. Marine diatoms have been microscopically studied for than a century whereas sequencing diatom species is a much more recent development (since the late 90s; e.g. [92, 93]).

## Conclusions and perspectives

The two methods are complementary. The strength of microscopy is its ability to identify a large majority of the taxa, bringing valuable ecological information based on the historical bibliographical data and ecological guild abundances. But, its weakness is imprecise identifications, particularly for the small taxa that were numerous in our samples, making comparison of turtles based on their epibiotic diatoms assemblages uncertain. An additional weakness is that microscopy requires well trained people to identify diatoms, and each sample needs to be examined for several hours under the microscope. If large sets of turtles were studied routinely, microscopy would be too demanding in terms of manpower.

On the other hand, metabarcoding enables the analysis of many samples at low cost (e.g. [94],). Sample treatments (e.g., extraction, PCR, sequencing, and bioinformatics) do not require rare experts in diatom taxonomy. Moreover also allows cryptic diatom diversity to be revealed, a difficult undertaking using microscopy. This allows better discrimination between turtles based on their epibiotic diatom assemblages. Therefore, we recommend using metabarcoding for massive comparisons of sea turtles based on their diatom assemblages. But these comparisons must be performed based on OTU abundances without taxonomic assignation because barcoding libraries are not yet complete enough. We discourage use of this methodology if ecological information is needed and if reference barcoding libraries do not cover sufficient marine diatom diversity.

## Supporting information

S1 Table *rbc*L alignment used for the constraint phylogeny in the Fig 6. (TXT)

S2 Table Spearman correlation coefficients calculated between the OTUs of Fig 7 and their abundances in the samples. (XLSX)
